# Clinical efficacy of extracranial-intracranial bypass for the treatment of adult patients with moyamoya disease: A protocol of systematic review of randomized controlled trials

**DOI:** 10.1097/MD.0000000000018211

**Published:** 2019-12-10

**Authors:** Jun Yang, Guang-fu Song, Hong-bin Li, Shi-hua Zhang, Fu-yi Yang

**Affiliations:** a Department of Neurosurgery; b Fourth Ward of Neurology Department; c Department of Gastroenterology; d Department of Neurology, First Affiliated Hospital of Jiamusi University, Jiamusi, China.

**Keywords:** efficacy, extracranial-intracranial bypass, moyamoya disease, safety

## Abstract

**Background:**

Moyamoya disease (MMD) is a major health concern associated with blocked arteries at the base of the brain. The aim of this study will synthesize the current evidence of the efficacy and safety of extracranial-intracranial bypass (EIB) for the treatment of adult patients with MMD.

**Methods:**

A systematically and comprehensively literature search will be performed in PubMed, EMBASE, Web of Science, CENTRAL, CINAHL, AMED, CBM, and CNKI to identify relevant randomized controlled trails (RCTs) investigating the efficacy and safety of EIB for treating MMD. We will search all above electronic databases from their inception to the July 30, 2019. Two review authors will independently perform study selection, data extraction, and conduct risk of bias evaluation using Cochrane risk of bias tool. We will also explore heterogeneity across studies. RevMan 5.3 software will be applied for statistical analysis performance.

**Results:**

This study will evaluate the efficacy and safety of EIB for the treatment of adult patients with MMD.

**Conclusion:**

The results of this study will provide latest evidence of the efficacy and safety of EIB for MMD.

**Dissemination and ethics:**

This study is based on published studies, thus, no ethical consideration is needed. The results of this study are expected to be published in peer-reviewed journals or will be presented on conference meeting.

**Systematic review registration:** PROSPERO CRD42019155839.

## 1 Introduction

Moyamoya disease (MMD) is a type of chronic and progressive steno-occlusive vasculopaty disease.^[1–4]^ It often occurs in East Asian populations, and results in some neurological diseases, such as ischemic or hemorrhagic stroke.^[5–8]^ It has been reported that it often attacks patients aged from 5 to 49 years old with female-to-male ratio of 2.2.^[9–11]^ Up to date, although its mechanisms are still fully elucidated, several genetic factors have found to be responsible for such condition.^[12–19]^

Extracranial-intracranial bypass (EIB) has been reported to mange patients with MMD.^[20–27]^ However, its efficacy and safety is still contrary, and no study has been conducted to assess its efficacy and safety systematically. Therefore, this study will systematically investigate the efficacy and safety of EIB for the treatment of patients with MMD.

## 2 Objective

The objective of this study is to explore the efficacy and safety of EIB for adult patients with MMD.

## 3 Methods

### 3.1 Study registration

This study has been registered in PROSPERO with CRD42019155839. It has been planned and reported based on the Preferred Reporting Items for Systematic Reviews and Meta-Analysis Protocol statement guidelines.^[28]^

### 3.2 Participants

We will consider patients with MMD aged 18 or older for inclusion with no limitation of sex, age, and race.

### 3.3 Interventions/exposure

The adult patients in the experimental group received EIB will be included.

The eligible individuals in the control group who received any treatments, except EIB will be considered.

### 3.4 Study types

We will consist of all randomized controlled trials (RCTs) on assessing the efficacy and safety of EIB for adult patients with MMD regardless language limitations.

### 3.5 Outcome measurements

The primary outcomes include cerebral blood volume, and incidences of cerebral hemorrhage and cerebral ischemia.

The secondary outcomes consist of mean transit time, time to peak, relative values of ischemic symptomatic hemispheres, quality of life, and any complications.

### 3.6 Literature search

We will search the following electronic databases of PubMed, EMBASE, Web of Science, CENTRAL, CINAHL, AMED, CBM, and CNKI to explore relevant randomized controlled trails (RCTs) investigating the efficacy and safety of EIB for treating MMD. This study will be performed from their inception to the July 30, 2019. All electronic databases will be searched without any language limitations. The full detailed search strategy for PubMed is showed in Table 1. Similar strategies will be adapted to the other electronic databases.

**Table 1:**
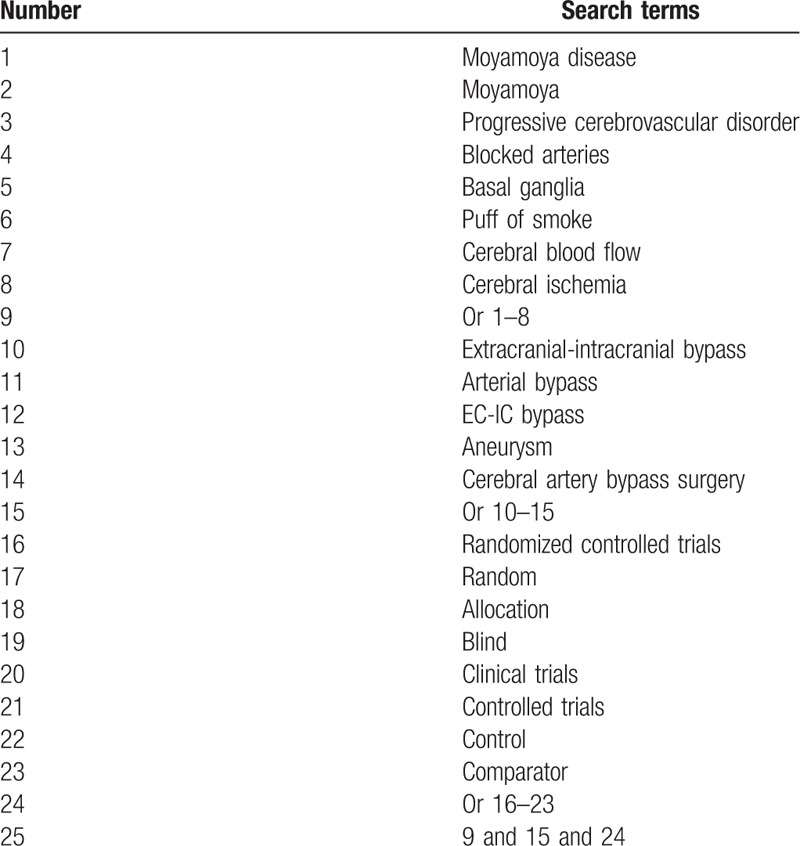
Search strategy sample used in PubMed database.

The reference lists of relevant studies and conference proceedings will also be searched to avoid missing any potential eligible studies.

### 3.7 Study selection

Two review authors will independently screen titles and abstracts of all literature records at first step. At the second step, full-texts of remaining studies will be read and investigated based on the inclusion criteria. Any disagreements between 2 reviewer authors will be solved by consensus or consultation of a third review author. The study selection process of this study will be exerted in a flowchart with specific exclusion reasons at different steps.

### 3.8 Data extraction and management

Two review authors will extract the following information from each eligible study using previous designed data extraction form: name of first author, published year, location, study setting, study design, patient characteristics, sample size, study methods, treatment details, outcomes, and safety. Any different opinions between 2 review authors will be settled down by a third review author through discussion. We will contact primary authors to obtain the information if it is missing, unclear, or insufficient. We will analyze available data if that missing data is not available.

### 3.9 Risk of bias assessment

Two review authors will independently assess the study quality for all eligible RCTs using Cochrane Risk of Bias Tool. This tool has 7 domains, and each item is further categorized into 3 risk of bias: low, unclear, and high risk of bias. Any disagreements between 2 review investigators will be solved by consensus or consultation of a third review author.

### 3.10 Measurement of treatment effect

As for continuous data, mean difference or standardized mean difference, and 95% confidence intervals will be calculated. As for dichotomous data, risk ratio and 95% intervals will be expressed.

### 3.11 Assessment of heterogeneity

We will use *I*^2^ test to explore the possible heterogeneity among eligible studies. The value of *I*^2^ ≤ 50% means reasonable heterogeneity. The value of *I*^2^ > 50% indicates significant heterogeneity.

### 3.12 Data synthesis

RevMan 5.3(Cochrane Community; city, London; country, England) software will be utilized for data synthesis and meta-analysis. If *I*^2^ ≤ 50%, we will use a fixed-effect model to pool the data, and will carry out meta-analysis. If *I*^2^ > 50%, we will use a random-effect model and will perform subgroup analysis. If there is still significant heterogeneity after subgroup analysis, we will not pool the data, and will report outcome results as a narrative summary.

### 3.13 Subgroup analysis

We will conduct subgroup analysis to investigate possible sources of heterogeneity across studies based on the different treatments, controls, and outcome measurements.

### 3.14 Sensitivity analysis

We will perform sensitivity analysis to identify the robustness of pooled outcome results by removing studies with low methodological quality.

### 3.15 Reporting bias

We will carry out funnel plot and Egger regression test to explore any possible reporting bias if sufficient eligible studies are included.

## 4 Discussion

To our best knowledge, no study has assessed the efficacy and safety of EIB for the treatment of adult patients with MMD. Thus, this study will be the first one to explore the efficacy and safety of EIB for adult patients with MMD. It will be carried out based on the comprehensively literature search and systematically data collection and analysis. The results of this study will summarize the most recent evidence for MMD treatment using EIB, and may provide helpful evidence for clinical practice.

## Author contributions

**Conceptualization:** Jun Yang, Guang-fu Song, Hong-bin Li, Fu-yi Yang.

**Data curation:** Jun Yang, Shi-hua Zhang, Fu-yi Yang.

**Formal analysis:** Jun Yang, Guang-fu Song, Shi-hua Zhang.

**Investigation:** Fu-yi Yang.

**Methodology:** Guang-fu Song, Hong-bin Li, Shi-hua Zhang.

**Project administration:** Fu-yi Yang.

**Resources:** Jun Yang, Guang-fu Song, Hong-bin Li, Shi-hua Zhang.

**Software:** Jun Yang, Guang-fu Song, Hong-bin Li, Shi-hua Zhang.

**Supervision:** Fu-yi Yang.

**Validation:** Jun Yang, Guang-fu Song, Hong-bin Li, Fu-yi Yang.

**Visualization:** Jun Yang, Shi-hua Zhang, Fu-yi Yang.

**Writing – original draft:** Jun Yang, Guang-fu Song, Hong-bin Li, Fu-yi Yang.

**Writing – review & editing:** Jun Yang, Guang-fu Song, Shi-hua Zhang, Fu-yi Yang.
